# Comparison of the effect of different surface treatments on the bond strength of different cements with nickel chromium metal alloy: An in vitro study

**DOI:** 10.4317/jced.53877

**Published:** 2017-07-01

**Authors:** Saumya Kapoor, Nayana Prabhu, Dhanasekar Balakrishnan

**Affiliations:** 1 Post graduate student; 2 Associate Professor, Department of Prosthodontics and Crown & Bridge; 3 Professor and Head , Department of Prosthodontics and Crown & Bridge

## Abstract

**Background:**

For success of any indirect metal restoration, a strong bond between cement and the intaglio surface of metal is imperative. The aim of this study is to evaluate and compare the effect of different surface treatment on the tensile and shear bond strength of different cements with nickel–chromium alloy.

**Material and Methods:**

120 premolars were sectioned horizontally parallel to the occlusal surface to expose the dentin. Wax patterns were fabricated for individual tooth followed by casting them in nickel chromium alloy. 60 samples were tested for tensile bond strength, and the remaining 60 for shear bond strength. The samples were divided into three groups (of 20 samples each) as per the following surface treatment: oxidation only, oxidation and sandblasting, or oxidation, sandblasting followed by application of alloy primer. Each group was subdivided into 2 subgroups of 10 samples each, according to the bonding cement i.e RM-GIC and resin cement. Samples were subjected to thermocycling procedure followed by evaluation of bond strength.

**Results:**

Two-way analyses of variance (ANOVA) was performed to compare the means of tensile and shear bond strength across type of surface treatment and cement, followed by post hoc parametric analysis. For all tests ‘*p*’ value of less than 0.05 was considered statistically significant.

**Conclusions:**

The surface treatment of oxidation and sandblasting followed by application of alloy primer offered the maximum tensile and shear bond strength for both RM GIC and resin cement. Resin cement exhibited greater tensile and shear bond strength than RM-GIC for all the three surface treatment methods.

** Key words:**Resin cement, resin modified glass ionomer cement, oxidation, sandblasting, alloy primer, tensile bond strength, shear bond strength, universal testing machine.

## Introduction

Indirect restorations are used in a variety of clinical situations, few of them are fabricated using metal alloys. Numerous factors are responsible for the long-term success of dental cast restorations. Choice of correct luting cement and surface condition of the intaglio surface of the fixed prosthesis, are important pre-requisites in achieving suitable retention for cast crowns or resin bonded restorations.

Adhesion of the cements to the intaglio surface of indirect restorations is imperative to durability of the restoration, particularly when there are difficulties during the restoration process due to reduced height of the tooth or improper taper of the preparation. Numerous superficial conditioning methods are present which alter the alloys surface and modify their morphological characteristics through chemical substances such as acids, by sandblasting, or by electrolytic deposition of ions chemically more reactive, promoting a greater affinity between the alloys and the cements, thereby increasing the retention and longevity of the fixed prosthesis.

The quality and strength of the dental cement is also a key factor which provides resistance to dislodgement of the prosthesis during function. Metal restorations are most commonly cemented with zinc phosphate and glass ionomer cement. However, the high solubility, reduced bonding to tooth structure and high stiffness associated with conventional cements has shifted the focus to resin based luting agents (1).

Self-adhesive resin cements offer high fracture strength, adequate bond to the dental structure and low solubility when exposed to oral fluids.

Resin-modified glass ionomer cement (RMGIC), has been developed by adding resin components to conventional glass ionomer cement (GIC), thus its mechanical and bonding properties are enhanced.

Bonding between resins and metals is a physicochemical phenomenon, resulting in mechanical interlocking of the cement with the metal surface and chemical interactions between the oxides present on the surface of metals and the carboxylic or phosphoric acid derivatives present in the cements (1).

Choice of correct luting cement and surface condition of the intaglio surface of the fixed prosthesis, are important pre-requisites in achieving suitable retention for cast crowns or resin bonded restorations. Therefore, the purpose of this study was to evaluate and compare the effect of different surface treatment on the tensile and shear bond strength of different cements with nickel–chromium metal alloy.

The Null hypothesis tested is that different luting agents and various surface treatments of the nickel chromium alloy have no effect on *in-vitro* tensile and shear bond strength values.

## Material and Methods

The study was approved by the Ethics Committee of Kasturba Medical College and Kasturba Hospital – Institutional Ethics Committee.

120 extracted, caries free, unrestored human maxillary first premolar teeth were used in the study. Storage of extracted teeth was done in 0.05% thymol solution to avoid microbial colonization (1). They were divided randomly into 12 experimental groups with 10 teeth in each group. All the procedures were carried out by one operator. Notches were made on the root surface to increase retention of tooth in acrylic resin. For preparation of acrylic resin blocks, self-cure acrylic resin powder and liquid (DPI – Rapid Repair, Mumbai) was mixed as per manufacturer instructions and poured in the aluminum die. The teeth were embedded 1mm above the cementoenamel junction in an acrylic resin while it was in the dough stage and allowed to completely set. The samples were then taken out of the aluminium die.

The acrylic blocks for tensile bond strength evaluation were of dimension 3x5cm, while those for shear bond strength were of 3x8cm.

A flat occlusal surface with dentin exposed was created at right angles to the straight-line axis of the tooth (2), using a diamond abrasive impregnated disk (3) in micromotor hand piece under copious water lubrication. A dental surveyor was modified and customized to receive the hand piece with the bur, to ensure a flat occlusal surface which is parallel to the horizontal plane.

The hand piece was fixed at that position moved back and forth across the occlusal surface of each tooth 20 times.

**Figure 1:**
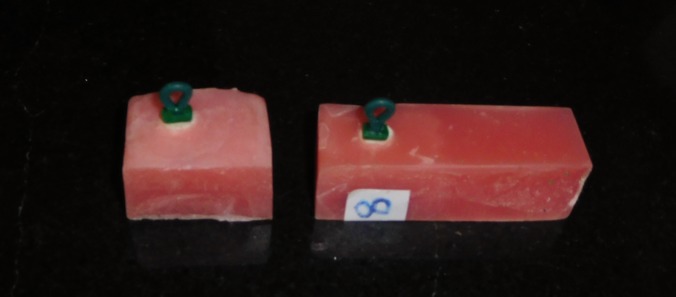
Fabrication of wax pattern on prepared teeth.

Wax patterns were prepared with casting wax (Star Wax CB) directly on the tooth surface. All the wax patterns prepared were of dimension 7x5mm (Fig. 1). An inverted ‘U’ shaped loop prepared using sprue wax of diameter 2mm (Bego,USA) was attached to the external surface of the patterns which were to be tested for tensile bond strength (2). Wax patterns were then invested and casted in nickel–chromium metal alloy (Wiron 99,Bego,USA) using an induction casting machine(OKAY PLUS, Galoni,USA). Castings were recovered from the investment. They were of same dimension, thereby standardizing the surface area of all the metal casting samples which will be bonded to the teeth samples. Total 120 castings were obtained respective to their tooth, 60 were tested for tensile bond strength and 60 for shear bond strength.

All the metal samples were oxidized. For oxidation treatment, the metal samples were exposed to temperature range of 600° - 980°C under vacuum, in the Multimat NTX press unit for 6 minutes 30 seconds to form an oxide layer (4). This was done to simulate the treatment which inadvertently occurs on the metal surface in case of a porcelain fused to metal restoration when ceramic firing is done for the ceramic facing.

The 60 nickel-chromium metal alloy samples to be tested for tensile bond strength, were divided into three groups of 20 samples each, according to the three surface treatments done i.e. oxidation only, oxidation followed by sandblasting treatment, oxidation, followed by sandblasting and chemical treatment with alloy primer.

The remaining 60 nickel-chromium metal alloy samples to be tested for shear bond strength, were similarly divided into three groups of 20 samples each, according to the three surface treatments done.

For sandblasting or airborne particle abrasion treatment, aluminum oxide was applied on the bonding surface of metal castings at 0.3 MPa pressure (70 psi) for 10 seconds at the distance of 10 mm (4).

After sandblasting treatment, the samples were cleaned under running tap water followed by ultrasonic cleaning in distilled water for 2 min.

Chemical surface treatment involved the application of metal primer (Alloy Primer) on the metal surface using applicator brush. It was then left to air dry for 20-30 seconds (4).

Each group was divided into two subgroups according to the cement used for cementing the metal casting to the tooth surface i.e. resin modified GIC (RelyX Luting 2) and a self-adhesive, resin- cement (RelyX U 200).

Cements were mixed at room temperature as per the manufacturer’s instructions and were placed within the working time recommended.

The cement used were the one available in clicker dispenser system, this ensured proper base-catalyst paste ratio.

For resin modified GIC (RelyX Luting 2), adequate amount of base and catalyst paste was dispensed in equal volumes (actual weight ratio is 1.3:1) and mixed into a homogenous mass of uniform colour within 20 seconds using a spatula. This mix was applied to the metal surface, which was then seated on the prepared tooth sample under firm finger pressure. Excess cement was removed from all sides after a brief exposure to blue light of intensity of 500mw/cm2 for 5 seconds per side (as per manufacturer instructions) (5). A firm finger pressure for 5 min was kept on the metal sample cemented to the teeth to allow complete setting after placement (5).

For resin cement (RelyX U200) adequate amount of base and catalyst paste was dispensed and mixed into a homogenous mass within 20 seconds using a spatula. This mix was applied to the metal surface, and seated on the prepared tooth sample under firm finger pressure. Excess cement was removed from all sides after a brief exposure to blue light of intensity of 500mw/cm2 for 2 seconds per side (as per manufacturer instructions) (5).

A firm finger pressure was kept on the metal samples cemented to the teeth for 5 minutes to ensure constant pressure distribution. The sample was light cured from all the four sides, with exposure time for each surface being 20 seconds (2).

All the samples were kept in distilled water at 37°C for 24 hours before subjecting them to thermocycling treatment (550 cycles at 55 degree Celsius and 5 degree Celsius with dwell period of 30 seconds - as per ISO TR 11450 standard) to simulate the oral environment conditions. Samples were again stored at 37°C for 24 hours before debonding.

In thermocycling, samples are subjected to temperature extremes occurring within the oral environment, this shows the relationship of the linear coefficient of thermal expansion between the tooth and material.

**Figure 2:**
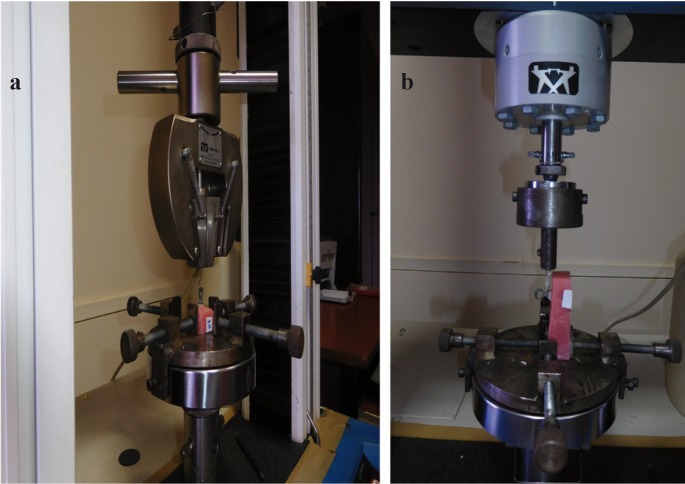
a) Measurement of tensile bond strength on universal testing machine. b) Measurement of shear bond strength on universal testing machine.

The measurement of tensile and shear bond strength was done on a universal testing machine (Instron 3366), (Fig. 2).

**Figure 3:**
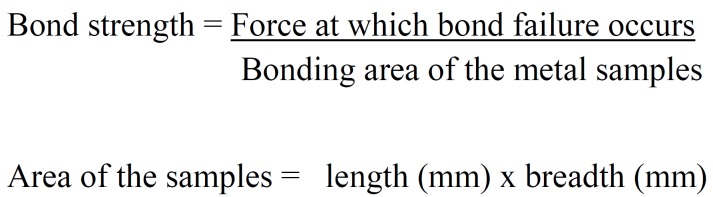
Formula.

The bond strength(MPa) was calculated by (1): (Fig. 3).

The data was analyzed using statistical package SPSS version 20. Mean and standard deviation were used to summarize tensile bond strength and shear bond strength. Two-way analyses of variance (ANOVA) was performed to compare the means of tensile and shear bond strength across type of surface treatment and cement used followed by post hoc parametric analysis.

## Results

 Table 1 and  Table 2 depict the mean and standard deviation values of tensile and shear bond strength in MPa for different surface treatments of the metal surface bonded with resin modified glass ionomer cement and resin cement.

**Table 1:**
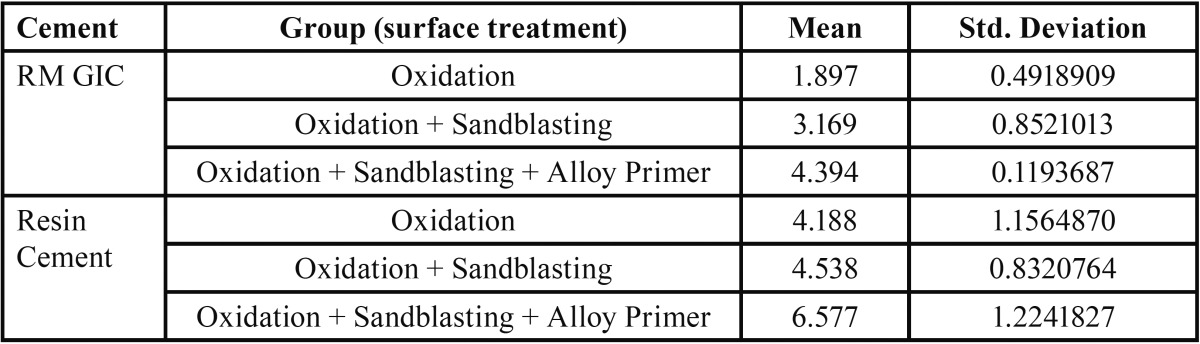
Mean value and standard deviation of tensile bond strength in MPa for different experimental groups (according to surface treatment) bonded with resin modified glass ionomer cement and resin cement. (n=10).

**Table 2:**
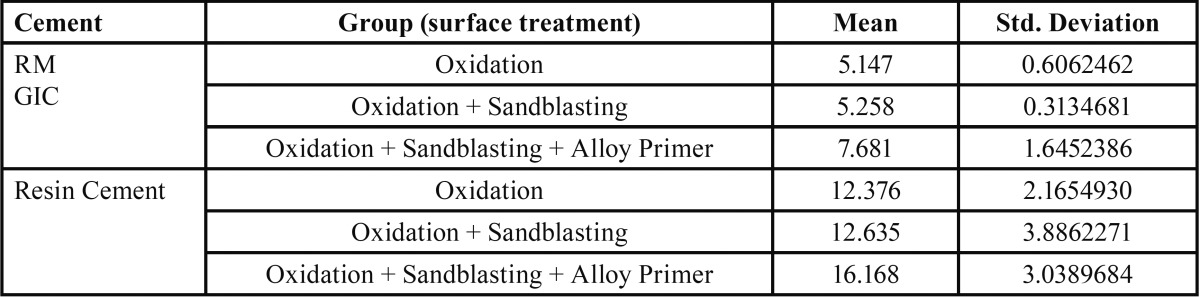
Mean value and standard deviation of shear bond strength in MPa for different experimental groups (according to surface treatment) bonded with resin modified glass ionomer cement and resin cement. (n=10).

Both RM-GIC and resin cement, exhibited maximum mean tensile and shear bond strength after the surface treatment of oxidation, sandblasting followed by application of alloy primer.

Two-way ANOVA was applied to observe the effect of surface treatment and cement on tensile and shear bond strength. No statistical interaction was observed between surface treatment and cements. But an association was observed between surface treatment 

(F2,56 = 11.983; *p* value < 0.001) and cement on the tensile bond strength (F1,56 = 168.994; *p* value < 0.001) as well as between surface treatment (F2,56 = 11,98; *p* value < 0.001) and cement on the shear bond strength (F1,56 = 168,9; *p* value < 0.001). A significant difference was observed in the tensile and shear bond strength between RM-GIC and resin cement (*p* value<0.001), with resin cement offering higher bond strength value.

For both the cements, a significant difference was observed in the tensile and shear bond strength between Group A and Group C, Group B and Group C (*p* value <0.001). The surface treatment which offered the maximum bond strength was combination of oxidation and sandblasting followed by application of alloy primer (Group C ) ( Table 3, Table 4).

**Table 3:**
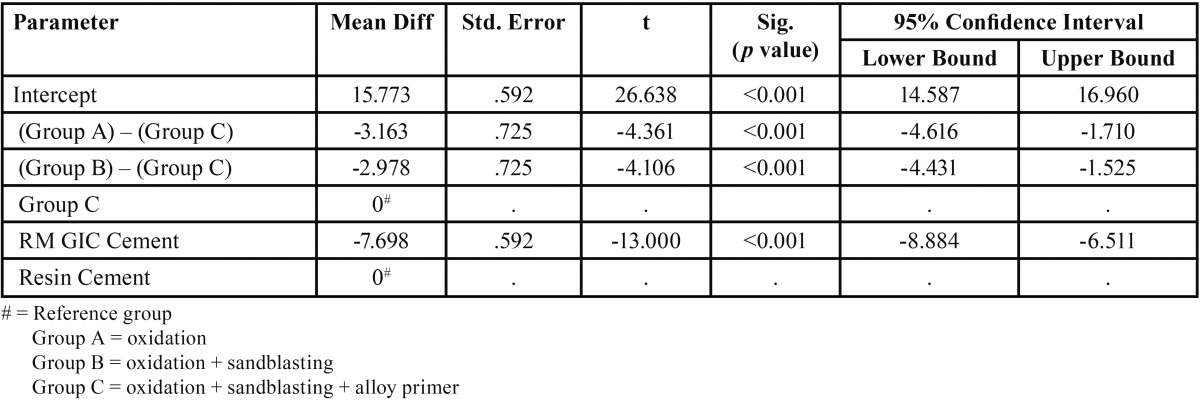
Results of post hoc comparisons of mean difference obtained through Two-way ANOVA Model for tensile bond strength.

**Table 4:**
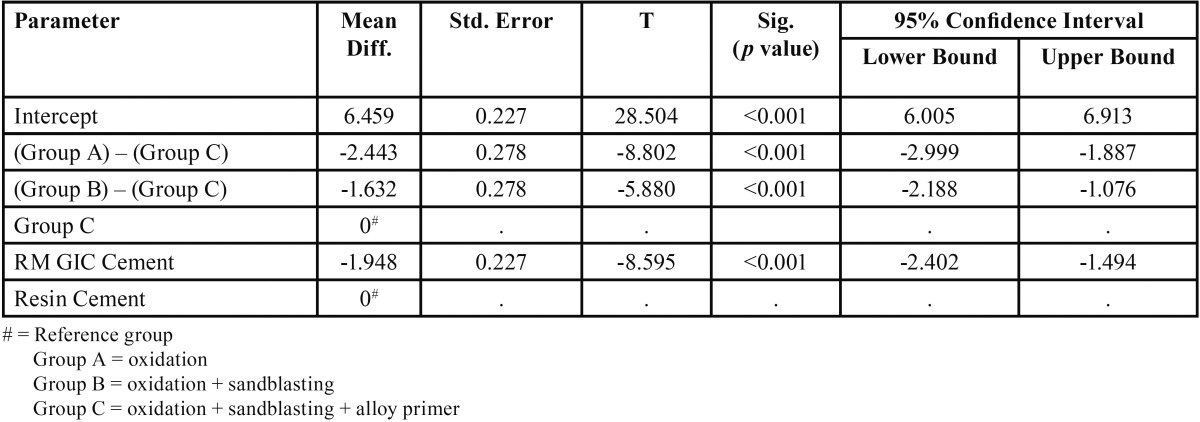
Results of post hoc comparisons of mean difference obtained through Two-way ANOVA Model for shear bond strength.

**Figure 4:**
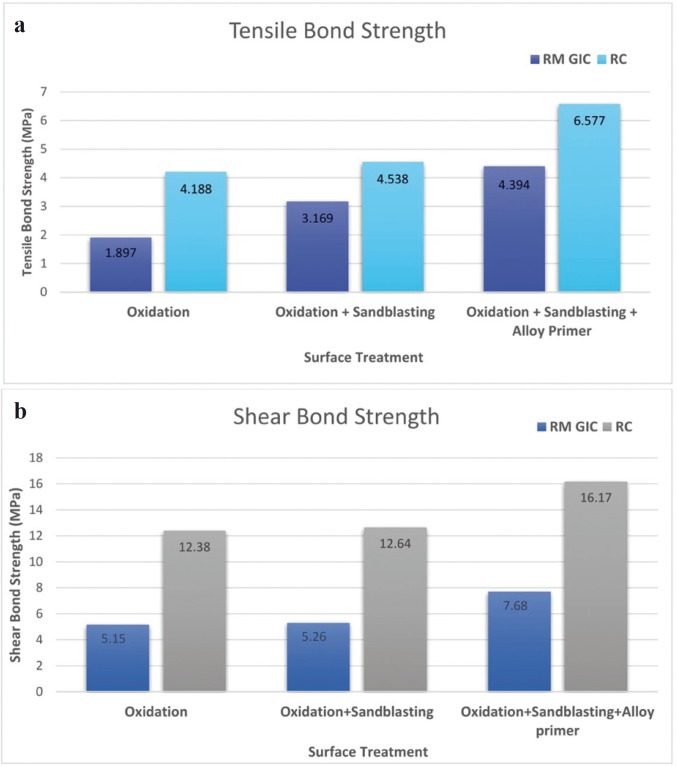
a) Bar chart depicting the comparison of tensile bond strength between RMGIC and resin cement after different surface treatments. b) Bar chart depicting the comparison of shear bond strength between RMGIC and resin cement after different surface treatments.

But no significant difference was found between Group A and Group B, for both the cements (Fig. 4).

## Discussion

Adhesive ability of luting agent is one of the key factors for longevity of any restoration. It is evaluated by tensile and shear bond tests, which were performed in this study.

The type of alloy used to fabricate the metal substructure of the crown may affect its retention. The choice of alloy in this study was Ni-Cr alloy. It is the most commonly used metal for making fixed adhesive prostheses. Nickel alloys are recognized for their ability to withstand the harsh oral environment and have a long-standing history of successful use in dentistry.

Base metals have higher free-surface energy and are more reactive than noble and high noble alloys, forming a thicker oxide layer. These oxides provide possible sites for chemical bonding and roughen the metal surface to provide some micromechanical retention (5). Rubo JH, Pegoraro LF (6) tested the tensile bond strength of a composite resin cement to various dental alloys. They achieved higher bond strength using base metal (NiCr, NiCrBe) than when using high noble alloy. Sen D, Nayir E, Pamuk S. (7) compared the tensile bond strength of high-noble, noble, and base metal alloys bonded to enamel. Lower bond strength to human enamel was observed with increasing nobility of an alloy, when using 2 types of resin cements. However, Abreu *et al.* (4) have reported that the type of metal does not affect the bond strength of resin cements.

The surface pre-treatments included in the study were oxidation only (Group A), oxidation and sandblasting (Group B), or oxidation, sandblasting followed by application of alloy primer (Group C).

For RM-GIC and resin cement, a significant difference was observed in the tensile bond strength between Group A and Group C, between Group B and Group C. Maximum tensile bond strength was noted in Group C. But no significant difference was found in the tensile bond strength between Group A and Group B.

Similarly, for shear bond strength a significant difference was observed between Group A and Group C, between Group B and Group C. Maximum shear bond strength was noted in Group C, for both RM-GIC and resin cement. But no significant difference was found in the shear bond strength between group A and Group B.

The surface treatment which offered the maximum tensile and shear bond strength for both the cements, was oxidation and sandblasting followed by application of alloy primer (Mean tensile bond strength for RMGIC = 4.394 MPa, Mean tensile bond strength for resin cement = 6.577 MPa), (Mean shear bond strength for RMGIC = 7.681 MPa, Mean tensile bond strength for resin cement = 16.168 MPa). Therefore, the null hypothesis was rejected.

These finding imply that the proposed chemical reaction produced between the NiCr alloy and the metal primer, effectively improves the bond strength of resin cement to base metal alloys. For surface treatment of oxidation, sandblasting followed by application of alloy primer, adherence was offered by both micromechanical means (due to oxidation and sandblasting) and chemical bonding due to alloy primer. Whereas, for surface treatment of oxidation or oxidation and sandblasting, bonding was due to micromechanical means only.

As per the manufacturer, the alloy primer contains MDP (10-methacryloyloxydecyl dihydrogen phosphate), an adhesive com-pound which contains phosphoric acid monomer components. These phosphoric acid monomers of MDP react with the metal oxides, while the double bonds on the other end of the molecule copolymerize with the resin cement monomers creating an enhanced metal cement bond (8,9).

These results agree with findings of Yoshida K, Taira Y, Matsumura H, Atsuta M (10) who evaluated the effect of adhesive metal primers on bonding a prosthetic composite resin to metals. They reported significant improvement in bond strength of noble and base metal alloys when these alloys were pre-treated using a metal primer before the cementation process. Francescantonio M, de Oliveira MT, Garcia RN, Romanini JC, da Silva NR, Giannini M. (11) evaluated the bond strength of resin cements to Co-Cr and Ni-Cr metal alloys using adhesive primers. They suggested that airborne-particle abrasion with alumina particles results in improved microtopography and possibly better wettability and penetration of the primers into the micro irregularities of the surface. Adhesion of resin to a substrate depends on both micromechanical interlocking and physicochemical bonding. Sandblasting with aluminum oxide provides the former, whereas the latter is due to the functional monomers present in resin-based materials and metal primers.

However, Parsa RZ, Goldstein GR, Barrack GM, LeGeros RZ (12) found a decrease in bond strength with the use of metal primers when compared to different surface pre-treatments applied to the same noble metal. Abreu A, Loza MA, Elias A, Mukho-padhyay S, Rueggeberg FA in 2009 (13) found no significant influence of metal type or surface pre-treatment on the bond strength of metal-ceramic copings cemented to minimally retentive standardized crown preparations.

Variations in the results reported by other authors might be caused by metal alloys and methodological differences among the studies, including the surface treatment protocol followed and the specific resin cement used

There are several luting cements, which bond both to tooth substance and the restorations. The literature has limited information about the comparison of the bonding performance of RMGIC and Self Adhesive Resin Cement to various fixed prosthodontic materials. For this study, two types of cements which have gained popularity in the last few years, RelyX Unicem 2 (Self Adhesive Resin Cement) and RelyX luting 2 (RMGIC), were chosen. Although these cements have different chemical components, they have similar applications. They are easy to use and are applied to the tooth without any pre-treatment i.e. etching, priming or bonding. They can be used with different prosthetic materials.

Thus, the other objective of this study was to evaluate which of the two cement has greater bond strength with NiCr alloy after the surface treatment of oxidation, sandblasting and application of alloy primer. A significant difference was observed between RM-GIC and resin cement for both tensile and shear bond strength (*p* value<0.05), with resin cement offering higher bond strength values (Mean tensile bond strength for RMGIC = 4.394 MPa, Mean tensile bond strength for resin cement = 6.577 MPa) , (Mean shear bond strength for RMGIC = 7.681 MPa, Mean tensile bond strength for resin cement = 16.168 MPa ).

Piwowarczyk *et al.* in 2004 (14) reported that RMGIC provided lower bond strength values with both metal alloy and ceramic than the self-adhesive resin cement which exhibited significantly higher values, as found in the present study. This can attribute to phosphoric-acid methacrylates in the self-adhesive resin cement, which provided a strong chemical reaction with MDP component of the metal alloy primer.

During porcelain firing procedures, an oxide layer is formed on the intaglio surface. Abreu *et al.* in 2009 (13)that this layer can be left in this state and directly cemented as the oxide layer provides for micromechanical retention (oxides roughen the surface) and a possible chemical reaction between the adhesive cement and the oxides.

Sandblasting is inexpensive and commonly used method to increase adhesion between metal and cement. After oxidation, the intaglio surface can also be airborne-particle abraded to provide for a clean and roughened surface prior to cementation. It increases cement wetting because of the mechanical removal of surface debris. This is in accordance with the previous study done by O’Connor *et al.* in 1990 (15) who concluded that retention of cast crowns can be improved by microblasting the internal surface.

But, for both the RM-GIC and resin cement, even though oxidation surface treatment exhibited the lowest bond strength, no statistically significant difference for shear bond strength was found between oxidised group and oxidation followed by sandblasting. This is in accordance with a study performed by Dixon *et al.* (16) who compared the shear bond strengths of two resin luting systems for metal alloy bonded to enamel, and found no significant difference between oxidised along with airborne particle abraded noble specimens and those that were only oxidized.

## Conclusions

Choosing a better combination of available cements and surface treatments to get a reliable bond, is an important concern in the dental research. In this study, it was observed that for both RM-GIC and Self-adhesive resin cement, the surface treatment which offered the maximum tensile and shear bond strength values was combination of oxidation, sandblasting and alloy primer. And for this surface treatment, compared to RM-GIC, self-adhesive resin cement offered stronger bond strength values.

Therefore, it can be concluded that the type of cement and the type of surface treatment of metal surface is an important and determining factor for bonding and retention of the prosthesis fabricated from NiCr alloy.

## References

[1] Bauer J, Costa JF, Carvalho CN, Souza DN, Loguercio AD, Grande RH (2012). Influence of alloy microstructure on the microshear bond strength of basic alloys to a resin luting cement. Braz Dent J.

[2] Hasti K, Jagadeesh HG, Patil NP (2011). Evaluation and Comparison of the Effect of Different Surface Preparations on Bond Strength of Glass Ionomer Cement with Nickel–Chrome Metal–Ceramic Alloy: A Laboratory Study. The Journal of the Indian Prosthodontic Society.

[3] Hattar S, Hatamleh MM, Sawair F, Al-Rabab'ah M (2015). Bond strength of self-adhesive resin cements to tooth structure. Saudi Dent J.

[4] Abreu A, Loza MA, Elias A, Mukhopadhyay S, Rueggeberg FA (2007). Effect of metal type and surface treatment on in vitro tensile strength of copings cemented to minimally retentive preparations. J Prosthet Dent.

[5] Silva BC, Salim MD, Kammer MG, Carvalho W, Gouvea DVC (2014). Influence of surface treatment on bond strength of resin cements to a nickel alloy. RGO, Rev Gaúch Odontol, Porto Alegre.

[6] Rubo JH, Pegoraro LF, Ferreira PM (1996). A comparison of tensile bond strengths of resin-retained prostheses made using five alloys. Int J Prosthodont.

[7] Sen D, Nayir E, Pamuk S (2000). Comparison of the tensile bond strength of high noble, noble, and base metal alloys bonded to enamel. J Prosthet Dent.

[8] Alloy Primer, Product brochure. Kuraray Co.

[9] Stefani A, Brito RB, Kina S, Andrade OS, Ambrosano GMB, Carvalho AA (2016). Bond Strength of Resin Cements to Zirconia Ceramic Using Adhesive Primers. J Prosthodont.

[10] Yoshida K, Sawase T, Watanabe L, Atsuta M (1995). Shear bond strengths of four resin cements to cobalt-choromium alloy. Am J Dent.

[11] Di Francescantonio M, de Oliveira MT, Garcia RN, Romanini JC, da Silva NR, Giannini M (2010). Bond strength of resin cements to Co-Cr and Ni-Cr metal alloys using adhesive primers. J Prosthodont.

[12] Parsa RZ, Goldstein GR, Barrack GM, LeGeros RZ (2003). An in vitro comparison of tensile bond strengths of noble and base metal alloys to enamel. J Prosthet Dent.

[13] Abreu A, Loza M, Elias A, Mukhopadhyay S, Loonet S, Rueggeberg F (2009). Tensile bond strength of an adhesive resin cement to different alloys having various surface treatments. J Prosthet Dent.

[14] Piwowarczyk A, Lauer HC, Sorensen JA (2004). In vitro shear bond strength of cementing agents to fixed prosthodontic restorative materials. J of Prosth Dent.

[15] O'Connor RP, Nayyar A, Kovarik RE (1990). Effect of internal microblasting on retention of cemented cast crowns. J Prosthet Dent.

[16] Dixon DL, Breeding LC, Hughie ML, Brown JS (1994). Comparison of shear bond strengths of two resin luting systems for a base and a high noble metal alloy bonded to enamel. J Prosthet Dent.

